# The role of SPATA2 in TNF signaling, cancer, and spermatogenesis

**DOI:** 10.1038/s41419-022-05432-1

**Published:** 2022-11-19

**Authors:** Valentina Masola, Nicola Greco, Pamela Tozzo, Luciana Caenazzo, Maurizio Onisto

**Affiliations:** 1 Department of Biomedical Sciences, University of Padova, Padua, Italy; 2 Department of Cardiac, Thoracic, Vascular Sciences and Public Health, University of Padova, Padua, Italy

**Keywords:** Cell biology, Cell signalling

## Abstract

The activation of TNF receptors can lead to cell death with a mechanism of cell necrosis regulated genetically and distinct from apoptosis which is defined as necroptosis. Necroptosis has been one of the most studied emerging cell death/signaling pathways in recent years, especially in light of the role of this process in human disease. However, not all regulatory components of TNF signaling have been identified in relation to both physiological and pathological conditions. In 2008, Spata2 (Spermatogenesis-associated protein 2) was identified as one of the seven fundamental genes for the cellular signaling network that regulates necroptosis and apoptosis. This gene had been cloned by our group and named Spata2 as its expression was found to be elevated in the testis compared to other tissues, localized at the Sertoli cell level and FSH-dependent. More recently, it has been demonstrated that deletion of Spata2 gene causes increased inhibin α expression and attenuated fertility in male mice. However, more importantly, five recently published reports have highlighted that SPATA2 is crucial for recruiting CYLD to the TNFR1 signaling complex thus promoting its activation leading to TNF-induced cell death. Loss of SPATA2 increases transcriptional activation of NF-kB and limits TNF-induced necroptosis. Here we will discuss these important findings regarding SPATA2 and, in particular, focus attention on the evidence that suggests a role for this protein in the TNF signaling pathway.

## Introduction

Inflammation is defined as an innate non-specific defense mechanism that starts following the presence of pathogens or damaged tissues and with the ultimate goal of eliminating the damage and starting the repair process. This process is locally mediated by some pro-inflammatory cytokines such as TNF and IL-1β that are produced by cells of the innate immune response. In particular, TNF is mainly produced by macrophages, though also by other types of cells including lymphoid cells, mast cells, endothelial cells, and fibroblasts. Most of the tissues and cells constitutively express TNF receptor 1 (TNFR1) which, once bound by TNF, triggers the formation of the TNFR1 signaling complex [1–3]. This protein complex (also named complex 1) requires specific adapter proteins such as TRADD and RIPK1 and, subsequently, the engagement of linear ubiquitin chain assembly complex (LUBAC), composed of the HOIL-1L, HOIP, and SHARPIN subunits. The processes of ubiquitylation and de-ubiquitylation are finely regulated and are crucial not only for the stability of TNFR1 but also for the fate of pro-inflammatory or pro-death TNF-dependent signaling. In particular, the deubiquitinases CYLD, which is also recruited to the complex, plays a fundamental role in attenuating the signaling pathway triggered by TNF [4]. The cellular signaling initiated by this complex can generate different responses such as the activation of NF-κB transcription factor or the mitogen-activated protein (MAP) kinase signaling which, in turn, goes towards regulating the pro-inflammatory and cell survival responses. Alternatively, under certain circumstances, a second complex (named complex 2) can form by recruiting RIPK1, FADD, and caspase-8 thus resulting in the induction of apoptotic cell death. If caspase-8 is inhibited, assembly following TNF stimulation promotes an alternative complex 2 leading to necroptotic cell death [4–6]. In this scenario, SPATA2 has recently appeared, identified by four independent studies as a new component of the TNFR1 signaling complex [7–10]. This review summarizes these findings and provides evidence that suggests a role for this protein in the TNF signaling pathway.

## SPATA2, TNF receptor 1 complex and cell death

In 2008, a study had already identified Spata2 as one of the seven fundamental genes for the cellular signaling network that regulates apoptosis and necroptosis, a regulated cellular necrosis mechanism discrete from apoptosis [11]. More recently, using mass spectrometry and co-immunoprecipitation methods, four studies have made it possible to identify SPATA2 as the protein capable of binding CYLD on one side and HOIP on the other and, therefore, to be enrolled as a new component of the TNFR1 signaling complex. Specifically, the ability of SPATA2 to interact with CYLD promotes the assembly of a complex consisting of a hetero-tetramer which then interacts with two LUBAC complexes by means of HOIP [9, 10, 12]. The hypothesis that SPATA2 is the connecting factor between CYLD and HOIP was further bolstered by the fact that recruitment of CYLD into TNF-Receptor Signaling Complex (TNF-RSC) was impaired in cells not expressing SPATA2 [9] as well as SPATA2 not being recruited to TNF-RSC in HOIP^−/^^−^cells [7].

**Fig. 1:**
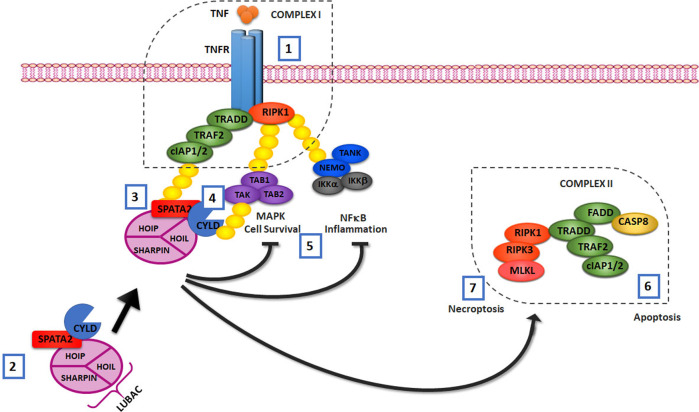
Role of SPATA2 at the level of TNF-RSC. After stimulation by TNFα (1), the CYLD/SPATA2/LUBAC complex (just pre-existing in the cytosol (2) is recruited into the receptor complex I through the interaction of LUBAC with the ubiquitin chains present in the complex (3). At the same time, the direct interaction of SPATA2 with CYLD results in an increase in the enzymatic activity of the latter which mediates the de-ubiquitylation (4) of some complex components such as RIPK1–thus playing a fundamental role in attenuating the signaling pathway triggered by TNFα. Therefore, activated CYLD attenuates NF-kB pro-inflammatory signaling and MAPK pro-cell-survival response (5). Alternatively, a second complex (named complex 2) can form by recruiting, among other factors, RIPK1, FADD, and caspase-8–thus resulting in the induction of apoptotic cell death (6). If caspase-8 is inhibited, assembly following TNF stimulation promotes an alternative complex 2 with MLKL leading to necroptotic cell death (7).

On the basis of this evidence, the activation and assembly model of the TNFR1 signaling complex may be updated as follows: after stimulation by TNF, the CYLD/SPATA2/LUBAC complex is recruited into the receptor complex through the interaction of LUBAC with the ubiquitin chains present in the complex. At the same time, the direct interaction of SPATA2 with CYLD results in an increase in the enzymatic activity of the latter (hydrolysis of polyubiquitin chains) thus suggesting that SPATA2 may be an allosteric activator of CYLD and not only a mere adapter for binding with HOIP [10, 12] (see Fig. 1).

In addition to this evidence, what effects did SPATA2 depletion have on the cellular phenotype? The most striking effect observed with Spata2^−/^^−^ cells was the reduction in cell death induced by TNF. In other words, it was found that the absence of SPATA2 greatly reduces the formation of the TNFR1 complex during treatment with TNF and, consequently, the processing of caspase-3 and necroptosis-apoptosis is decreased [9, 10].

More recently, the protective and anti-inflammatory roles of Spata2 have also been confirmed. Yang XD et al. demonstrated and defined a centrosomal SPATA2/CYLD-PLK4 signaling axis that suppresses NLRP3 inflammasome activation suggesting that SPATA2 was a negative regulator of inflammasome activation and, thus, of inflammation. Specifically, SPATA2/CYLD complex is responsible for PLK4 deubiquitination which, in turn, facilitates NEK7 phosphorylation and reduction of NLRP3 activation [13]. In summary, the role of SPATA2 in the inhibition of inflammasome depends on its capacity to activate CYLD which, via PLK4/NEK7, impairs NLRP3 activation.

Confirming this role and using a transient focal cerebral ischemia/reperfusion animal model, Ren et al. showed that SPATA2 expression was reduced in rat brains after I/R and that Spata2 knockdown caused an increase in microglia cells with a consequent increase in the expression of TNF, IL -1β and IL-18. Furthermore, the silencing of Spata2 resulted in the activation of p38 MAPK and NLRP3 inflammasome with consequent activation of NF-κB signaling [14].

SPATA2 also participates in the regulation of complex II even if the exact molecular mechanism of its action has not been completely determined. So far, it is known that complex II formation is compromised in the absence of SPATA2 [9]. Since SPATA2 enhances the deubiquitination activity of CYLD [9, 10], and CYLD had been shown to deubiquitinate RIPK1 [15], the presence of SPATA2 can promote TNF-induced cell death.

Overall, this experimental evidence highlights for the first time the role played by SPATA2 in TNF-induced cell death and hypothesizes a functional link between CYLD and SPATA2 for signaling through the TNFR1 complex I (functional for the inflammatory response) and for the assembly of the TNFR1 complex II (functional for necroptosis/apoptosis) (see Fig. 1).

As suggested by some authors, however, the picture may not be complete since not all the functions of SPATA2 in promoting cell death can be explained on the basis of its interaction with CYLD at the level of TNF receptor complexes [12, 16]. Further investigations will therefore be necessary to demonstrate whether or not SPATA2 can interact with other proteins at the level of TNFR1 signaling complex.

## SPATA2, TNF receptor 1 complex, and spermatogenesis

Male infertility is a primary health problem that, according to the World Health Organization, refers to the inability of the male partner to cause pregnancy in a clinically normal female [17]. While Y chromosome-linked azoospermia factors have received a great amount of investigation, they are likely to account for only a small proportion of genetically based male infertility [18].

**Fig. 2:**
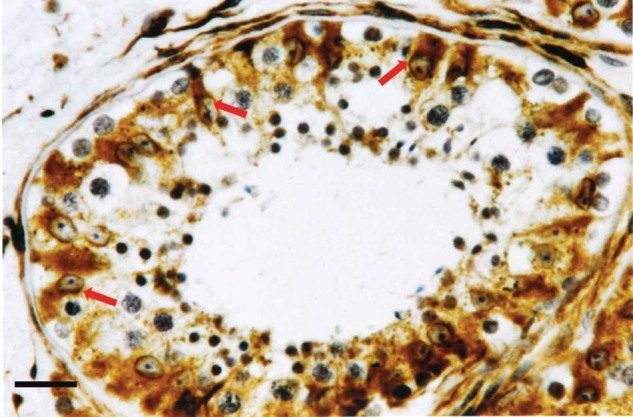
Immunohistochemical localization of SPATA2 at the Sertoli cell level. The expression of SPATA2 is highlighted as a brown color that is localized in correspondence with the cytoplasm of Sertoli cells (indicated by arrows) which are easily recognizable by their elongated and branched shape and by an ovoid nucleus with the evident nucleolus. No positivity was observed in any cell of the spermatogenic germline. Scale bar = 20 μm.

In an attempt to isolate new spermatogenesis-associated genes, some years ago we cloned from a human testis cDNA library a novel sequence named Pd1 (later *SPATA*2) and submitted it to GenBank under accession no. U28164. Pd1 cDNA sequence is 2.7 kb long and encodes for a 520-amino acid protein with a predicted molecular weight of 58.4 kDa [19, 20]. Immunohistochemical analysis on sections of human testicle revealed the presence of PD1 at the Sertoli cell level (Fig. 2) as well as various levels of its expression in different testiculopathies suggesting that PD1 protein production by Sertoli cells seems to be under the influence of spermatogenic cells [19]. To further elucidate the role of this gene in the regulation of spermatogenesis, we cloned the rat ortholog of Pd1 and studied its expression. We have shown that the expression of this protein is sensitive to FSH and that its mRNA levels in rat testis increase with advancing age through adulthood. [21]. These results suggest the involvement of this protein in the FSH-dependent function of Sertoli cells confirming the hypothesis that PD1 could play a role in the regulation of spermatogenesis. According to the Gene Nomenclature Committee, the name SPATA2 (Spermatogenesis-associated protein 2) was proposed for this protein.

In the meantime, a further and definitive confirmation of the role of SPATA2 in the control of correct spermatogenesis has come from a recent article in which the results of the deletion of *Spata2* by CRISPR/Cas9n in male mice were illustrated [22].

As regards the phenotypic analysis of the reproductive system, in the Spata2^−/−^ mice it was shown that the testes of the 120-day knockout mice had a decrease of almost 40% in size and weight. In addition, changes in the histo-morphology of the seminiferous epithelium emerged with a 40% reduction in the number of spermatozoa while the proliferation of germ cells in the seminiferous tubules was reduced by 28% despite the presence of an unchanged number of Sertoli cells. Finally, it was shown that the deletion of *Spata2* led to an increase of about 70% of the alpha subunit of inhibin in the testes which was accompanied by a decrease in expression for FSHb in the pituitary gland [22] which plays a pivotal role in the regulation of spermatogenesis [23]. Overall, these data confirmed the impact of SPATA2 on male fertility suggesting that SPATA2 ensures the normal secretory function of Sertoli cells. At the molecular level, this role can be explained in light of those discoveries that have identified SPATA2 as an essential component for the assembly of the TNFR1 signaling complex and, therefore, for the response to TNF [7–10]. Considering that Sertoli cells are involved in maintaining immune privilege within the testis [24] and that TNF is involved in the regulation of physiological and inflammatory processes in that organ by activating the transcription factor NF-κB [25], the finding that SPATA2 modulates NF -κB signaling [9] may be consistent with the hypothesis that high levels of SPATA2 expression play a role in the immune privilege of the testes and in the control of inflammation and/or cell death on the inside. In addition, it has recently been demonstrated that RIPK1 and RIPK3, as well as the RIPK3 substrate MLKL, cause programmed necrotic cell death in male mouse reproductive organs. Moreover, both Ripk3- and Mlkl-knockout mice retain “youthful” morphology and function into advanced age, while those of age-matched wild-type mice deteriorate [26, 27].

Since RIPK1-RPK3-MLKL complexes are important downstream members of the TNF signaling pathway leading to necroptosis and SPATA2 is involved in this phenomenon via LUBAC and CYLD, we can surmise that high levels of SPATA-2 could contribute to the aging-associated necroptosis inside the testis.

## SPATA2, TNF receptor 1 complex, and cancer

It has now been established by various epidemiological studies that chronic inflammation predisposes to various forms of cancer. This link that unites inflammation and cancer has been defined as “cancer-related inflammation” (CRI) and according to some authors it can be counted in all respects among the “hallmarks” of cancer [28, 29]. In fact, an inflammatory component is found in the tumor microenvironment of most of the neoplastic tissues and, in particular, it refers to the infiltration of white blood cells and tumor-associated macrophages (TAM) [30]. An equally important role is given to the presence of inflammatory mediators such as TNF, IL-1, IL-6, and chemokines, such as CCL2 and CXCL8 [31].

Since SPATA2 has been identified as a TNFR1 modulator required for TNF-induced inflammation and necroptosis [12, 14], some authors have attempted to study the expression of *SPATA2* and *TNFA* in the tissues of 171 patients with low-grade serous ovarian cancer (LGSOC), high-grade serous ovarian cancer (HGSOC), endometrioid and clear cell ovarian cancer (OC) compared to 28 non-malignant control tissues [32]. The expression of *TNFA* and *SPATA2* was found to be significantly higher in OC than in control tissues. In grade 2 and 3 tumors, *SPATA2* was expressed more than in grade 1 tumors as well as in HGSOC compared to LGSOC. Kaplan–Meier survival analyses showed that patients with ovarian tumors with high *SPATA2* expression were associated with reduced progression-free survival and overall survival. Furthermore, pro-inflammatory stimuli such as TNF and IL-1β significantly increased the expression of SPATA2 in ovarian cancer cell lines. This increased SPATA2 expression in some types of cancers could be explained by the presence in its promoter region of several binding sites for transcription factors such as NF1 [33] which plays an important role in the regulation of cell growth and in tumorigenesis [34]. In a subsequent study, the same authors analyzed the tumor expression of TNF and its regulator SPATA2 in relation to the clinical-pathological characteristics and clinical outcome of patients with endometrial carcinoma (EC) [35]. They were thus able to demonstrate that TNF and SPATA2 are always significantly higher in EC tumor samples than in non-malignant control tissues. Furthermore, a high expression of both markers is associated with reduced recurrence-free survival and overall survival. On the whole, the study showed that elevated TNF and SPATA2 mRNA expressions are independently associated with poor prognoses in patients with EC.

Although closely linked to the expression of TNF in the tumor context, these data suggest a role of SPATA2 in tumorigenesis which will necessarily have to be confirmed by further investigations into other neoplasms.

## Conclusion

More than twenty years after the cloning and discovery of SPATA2, we can safely say that what we had hypothesized at the time about the possible function of this protein in the control of spermatogenesis has been confirmed by several other studies over the years. Above all, Zhao’s important experimental work shows that in the knock-out mice the lack of SPATA2, a protein that is expressed at high levels in Sertoli cells, causes a reduction in testicular mass and in the number of spermatozoa with an attenuation of the reproductive capacity in the male [22]. Overall, this evidence confirms that SPATA2 is essential to maintaining spermatogenesis at normal levels.

In parallel, over the last few years, several proteomic analysis and immunoprecipitation studies have made it possible to identify in a precise and mechanistic way Spata2 as a new actor that contributes to TNF signaling by interacting with LUBAC and CYLD at the level of the TNF receptor signaling complex. In this context, the loss or lack of SPATA2 results in an increase in the transcriptional activity of NF-κB and in a limitation of cell death induced by TNF. In a nutshell, and as suggested by Feltham et al. [16], the role of SPATA2 could be defined as follows: “Keeping the TNF signal short and sweet”. Nonetheless, some authors suggest that SPATA2 may have CYLD-independent functions by hypothesizing that SPATA2 may interact with other proteins in the TNF receptor complex under conditions that lead to cell death [9, 12]. Although the picture of the situation is now much clearer and more delineated, further characterization of the role of SPATA2 could help shed light on the complicated and crowded TNF receptor scene.

## Data Availability

Data sharing is not applicable to this article as no datasets were generated or analyzed during the current study.
